# Brief mock-scan training reduces head motion during real scanning for children: A growth curve study

**DOI:** 10.1016/j.dcn.2023.101244

**Published:** 2023-04-13

**Authors:** Peng Gao, Yin-Shan Wang, Qiu-Yu Lu, Meng-Jie Rong, Xue-Ru Fan, Avram J. Holmes, Hao-Ming Dong, Hai-Fang Li, Xi-Nian Zuo

**Affiliations:** a College of Information and Computer, Taiyuan University of Technology, No. 79 West Street Yingze, Taiyuan, Shanxi 030024, China; b State Key Laboratory of Cognitive Neuroscience and Learning, Faculty of Psychology, Beijing Normal University, No 19 Xinjiekouwai Street, Haidian District, Beijing 100875, China; c Developmental Population Neuroscience Research Center, IDG/McGovern Institute for Brain Research, Beijing Normal University, No 19 Xinjiekouwai Street, Haidian District, Beijing 100875, China; d Institute of Psychology, Chinese Academy of Sciences, No 16 Lincui Road, Chaoyang District, Beijing 100101, China; e Department of Psychology, Yale University, 1 Prospect Street, New Haven, CT 06511, USA; f National Basic Science Data Center, No 2 Dongsheng South Road, Haidian District, Beijing 100190, China

**Keywords:** mock scan, head motion, growth curve, pediatric chart, quality control, neurodevelopment, resting-state fMRI

## Abstract

Pediatric neuroimaging datasets are rapidly increasing in scales. Despite strict protocols in data collection and preprocessing focused on improving data quality, the presence of head motion still impedes our understanding of neurodevelopmental mechanisms. Large head motion can lead to severe noise and artifacts in magnetic resonance imaging (MRI) studies, inflating correlations between adjacent brain areas and decreasing correlations between spatial distant territories, especially in children and adolescents. Here, by leveraging mock-scans of 123 Chinese children and adolescents, we demonstrated the presence of increased head motion in younger participants. Critically, a 5.5-minute training session in an MRI mock scanner was found to effectively suppress the head motion in the children and adolescents. Therefore, we suggest that mock scanner training should be part of the quality assurance routine prior to formal MRI data collection, particularly in large-scale population-level neuroimaging initiatives for pediatrics.

## Highlights

• Young children aged from 6 to 9 years benefit the most from mock scanner training.
• A 5.5-minute mock scan training greatly improves the performance in formal scans hereafter.
• The 93*.*5th percentile from pediatric charts is recommended for resting-state fMRI motion quality control.

## Graphical Abstract

**Figure:**
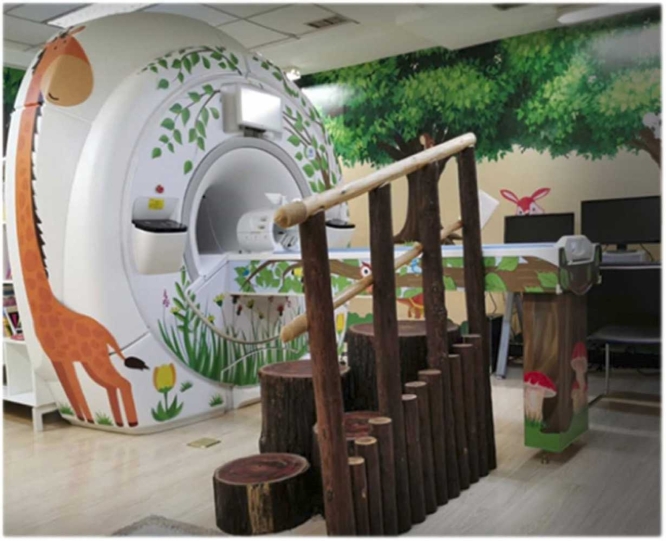

## 1 Introduction

The human brain and behavior are characterized by a series of dynamic changing processes across the lifespan, especially in childhood and adolescence. Understanding of developmental changes in neural mechanisms and the associations with cognition and behavior is the fundamental goal in the field. To achieve this, large-scale consortium projects in developmental populations, such as the IMAGEN Project (Mascarell Maričić et al., 2020), Adolescent Brain Cognitive Development (ABCD) (Bjork et al., 2017), Lifespan Human Connectome Project (Van Essen et al., 2013), Human Connectome Project-Development (Harms et al., 2018), Healthy Brain Network Biobank (Alexander et al., 2017), Brain Imaging Data Exchange (Di Martino et al., 2014), and Chinese Color Nest Project (Liu et al., 2021), have been recently initiated around the world. In these studies, MRI is widely employed to assess brain anatomy and function. However, although standard pipelines in data collection and preprocessing were proposed to improve data quality, head motion during scanning remains a nontrivial issue that needs to be directly addressed when interpreting MRI results.

Head motion refers to nonvoluntary head movements during MRI scanning, and has been widely recognized as a nuisance factor distorting MRI observations. Heavy head motions cause severe artifacts in anatomical scans, including blurring of the gray-white matter interface, leading to incorrect tissue segmentation (Caballero Gaudes and Reynolds, 2017, Savalia et al., 2017) and the underestimation of cortical thickness and volume (Alexander Bloch et al., 2016; Pardoe et al., 2016; Savalia et al., 2017). In functional MRI (fMRI) studies, head movement weakens long-range connectivity estimates while increasing short-range connectivity estimates (Poldrack et al., 2002, Dijk et al., 2012), and similar results have been reported in adolescents with several functional connectivity (FC) metrics (seed-based FC and the amplitude of low-frequency fluctuation (ALFF)) (Satterthwaite et al., 2012). High-motion datasets exhibit more pronounced negative motion–activation relationships; conversely, low-motion datasets exhibit more pronounced positive motion–activation relationships (Cgyab et al., 2013). Correlation of head motion indicates reduced distant functional connectivity, primarily in the default mode network, in individuals who had a large amount of head motion (Zeng et al., 2014). In quantitative MRI analysis, even small-amplitude head micromovements in a single data frame can induce systematic distortions (Ciric et al., 2017). Reported group differences in clinical studies, especially resting- state FC-based studies, may possibly reveal artificial effects rather than actual neurological factors if head motion is not strictly controlled (Cgyab et al., 2013, Dijk et al., 2012). Therefore, fMRI data with limited head motion during image acquisition are required to ensure the reliability and validity of the subsequent analysis.

Converging evidence supports the idea that head motion varies greatly across participant populations. Children and adolescents in particular can become anxious during data collection due to their unfamiliarity with the MRI machine, the presence of loud noises, and discomfort from lying within a narrow bore, which is a possible reason that children show the highest head motion during MRI collection relative to other age groups (Dosenbach et al., 2017). Compared with teenagers, adults show more random head motions, and young adults move more slowly and rhythmically. Meanwhile, older adults tend to have more head motion than younger adults (Pardoe et al., 2016, Savalia et al., 2017). This phenomenon results in potentially exaggerated differences in brain activity and structure between age groups (Bullmore et al., 2015, Poldrack et al., 2002, Power et al., 2014, Power et al., 2019, Satterthwaite et al., 2012). Other studies have also provided evidence that age-related differences in cortical thickness may be exaggerated by age-related differences in head motion (Salat et al., 2004) and that the increasing disparities between various age groups are also affected by head motion (McKay et al., 2014). Although larger head motion is reported in teenagers, a single threshold derived from the young adult population is often applied across age groups, which can result in massive data loss after quality control (Carolina et al., 2019, Zuo et al., 2019). The extent to which head motion varies with age and whether a single head motion criterion works for every point in the lifespan remain to be systematically investigated.

A number of metrics have been developed to quantify head motion. The spatial standard deviation of successive difference images, DVARS, is a whole-brain measure of the temporal derivative of image intensity by obtaining the root mean square variance across voxels. It is close to other statistical methods of estimating noise variance in the presence of drift (Poldrack et al., 2002, Smyser et al., 2011). Framewise displacement (FD) is another frequently utilized metric that accesses the absolute or root mean square movements along three translational and three rotational axes. FD represents the overall effects of subtle in-scanner movements from volume to volume, and it is closely related to motion artifacts, making it a much more useful estimate of problematic motion (Cgyab et al., 2013, Dijk et al., 2012, Satterthwaite et al., 2012). Although a criterion of 0.2 mm FD (Power et al., 2012) has been applied as a general threshold for quality assurance, widely used alternative thresholds have been used in other studies (Frew et al., 2022, Jolly et al., 2020). Several strategies have been proposed to address motion artifacts (Ciric et al., 2017), for example, the Friston 24-parameter (6 head motion parameters, 6 head motion parameters one time point before, and the 12 corresponding squared items) model (Friston et al., 1996), which regresses out head motion-related nuisance directly from fMRI time series. The motion scrubbing method removes time frames with heavy motion and then concatenates the temporal adjacent time points (Van Dijk et al., 2010). Independent components analysis (ICA) is also utilized to identify motion-related components, such as ICA-fix (Salimi Khorshidi et al., 2014) and ICA-AROMA (Pruim et al., 2015); the former requires manual labeling, while the latter automatically classifies nuisance components and is then regressed out from the time series. Another simple but effective strategy, global signal regression (Murphy and Fox, 2017) is also widely used to reduce the motion effect and has proven to be helpful in identifying network organization. However, head motion can also present as involuntary physical activity and reflects heritable traits (Zeng et al., 2014), algorithm-based strategy only diminishes the effects. Accordingly, priority should be placed on the improvement of the data quality at the initial stage of data collection.

Researchers have made some progress in exploring physical techniques to reduce head movements, such as administering a physical anesthetic or using head fixation to limit head movement during data collection, which is considered to be efficient for short scan durations (Bookheimer, 2000, Kotsoni et al., 2006, Poldrack et al., 2002, Davidson et al., 2003). Tactile feedback with medical tape is another effective strategy to restrain head motion (Krause et al., 2019, Jolly et al., 2020). However, these methods are rarely adopted in pediatric neuroimaging because it is less comfortable for a child lying in the MRI machine. Movie watching is suggested to be an efficient way to significantly reduce head motion in participants younger than 10 years of age, with a more than 70% reduction in mean FD (Greene et al., 2018). Performing a practice session in a mock scanner that simulates an MRI setting is another popular technique (Raschle et al., 2009, Slifer et al., 1993). The mock scanner acts as a device that plays the scanning sound and simulates a true scanning environment, featuring hardware and profiles that can be comparable to the real scanner. Before performing a formal scan session, the participant is provided with behavioral training to help them become accustomed to the magnetic field’s noise and reduce their discomfort (Rosenberg et al., 1997). This will largely help them feel more at ease inside the scanner and focus better on the experimental task. Following scanner training, young children (4–6 years old) present with less overall movement and are better able to adapt to the scanning environment, which increases the experiment’s rate of success (Hallowell et al., 2008, Slifer et al., 1994). It merits additional investigation and debate as to whether mock scanner training significantly reduces children’s and adolescents’ head movement. With the rapid accumulation of pediatric neuroimaging datasets, it has become a popular strategy, and ABCD has already set up mock scanner training in their data collection pipeline (Casey et al., 2018).

In this study, by leveraging the Chinese Color Nest Project (CCNP), an accelerated longitudinal neuroimaging dataset (Liu et al., 2021), we aim to provide some guidance for applying mock scanner training to reduce head motion-related issues in children and adolescent populations. First, developmental curves were constructed to depict the age-related variations in head motion with mock scanner data. Next, we evaluated the effectiveness of a short-term mock training before formal MRI scanning in children and adolescents. Finally, to systematically depict how head motion varies from children through adolescents and into young adulthood, we used a group of young adult data from the ’Imaging Chinese Young Brains (ISYB) dataset (Gao et al., 2022). We hope our findings can clarify the extent to which a single head motion criterion works across development, providing recommendations on how to balance between sample sizes and possible ‘aggressive’ restrictions on head motion in children and adolescents.

## 2 Materials and methods

### 2.1 Participant recruitment

All head motion data included in this study were taken from an accelerated longitudinal database, the Chinese Color Nest Project (CCNP: https://www.scidb.cn/en/c/ccnp) which is designed for assessing the developmental process of the brain and behavior after 6 years of age (Liu et al., 2021, Zuo et al., 2017). Acceleration was obtained through a mixture of cross-sectional and longitudinal designs to achieve long-term follow-up studies, such as lifespan development cohorts (Nooner et al., 2012, Thompson et al., 2011). Each participant was a Chinese Han person of school age who had neither neurological nor mental health issues nor had ever used psychotropic medication and underwent scanning 3 times with a 1.25-year interval (Liu et al., 2021, Dong et al., 2020, Dong et al., 2021). School-age typically developing children were recruited at two sites: The Institute of Psychology, Chinese Academy of Sciences in Beijing (CCNP-PEK), and Southwest University in Chongqing (CCNP-CKG). A total of 319 participants (CCNP- CKG: n = 196; CCNP-PEK: n = 123) were included in the current study, consisting of 578 MRI scans (CCNP-CKG: n = 419; CCNP-PEK: n = 159). Wechsler Intelligence Scale for Children intelligence quotient (IQ) standard scores below 80, a history of neurological or mental disorders, a family history of such disorders, organic brain diseases, physical contraindications to MRI scanning, and a total Child Behavior Checklist T score higher than 70 were excluded from further analysis. Each subject was asked if they had a fear of isolation or a problem with laying up before formally enrolling in the study, and all participants and their parents/guardians provided written informed consent. To ensure that the scan went well, children who exhibited the aforementioned symptoms were excluded from the study. The collaborating institutions’ review committees gave their approval for the current study (Institute of Psychology, Chinese Academy of Sciences, and Southwest University). All participants and their parents/guardians gave written informed consent before participating in the study.

### 2.2 Mock scanner training

**Fig. 1:**
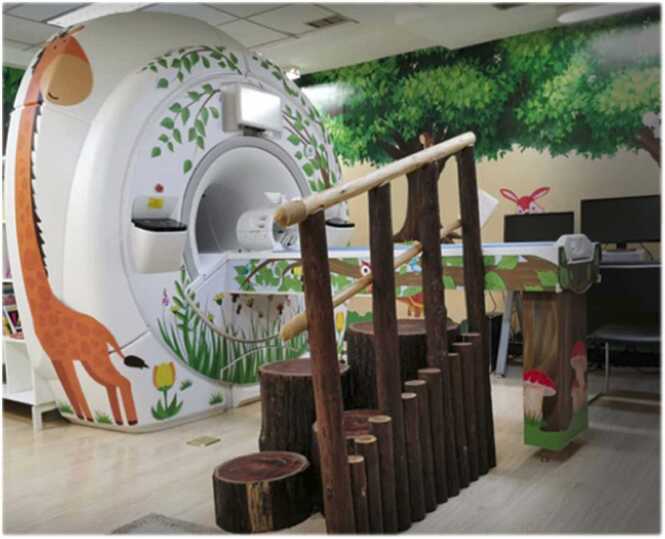
The mock scanning facility in the Magnetic Resonance Imaging Research Center at IPCAS.

A mock scanner (MoTrak Head Motion Tracking System; Psychology Software Tools), a 1:1 model of the GE MR750 3 T MRI scanner, is in use at the CCNP-PEK (Fig. 1), which simulates the real scanning situation without the magnetic filed and radio frequency pulse. It helps the participant adapt to the narrow cavity and RF pulse noise before the formal MRI scan and is believed to effectively relieve the anxiety and improve head motion control. The mock scanner consists of the main console, transmitter, and sensor. The sensor, which is worn on the participant’s head, determines the position of the head in space relative to the transmitter. The sensor records angular rotations as well as positional displacements from an initially calibrated position. This information is displayed and logged by the program in real time, allowing observation of head motion in an MRI simulator. In addition, the mock scanner’s cartoon sticker decorations create a child-friendly environment. To achieve high-quality scans with little head motion, the scanning at the CCNP-PEK site was performed as follows:

• The child took off his or her shoes and sat on the mock scanner bed, while the basic matters (keep his or her head still and don not fall asleep) and the experimental task process (watch a movie) were explained, and the head motion detection belt were affixed.
• The child was asked to lie on the bed, and the position of the helmet and cushion were adjusted. The child was asked how he or she felt to ensure it was comfortable for the child’s body.
• For young children (< 6 years old), their parents were asked to assist in pacifying the child. The children and parents were informed that they should not move or speak during the whole scanning process. Parents were allowed to touch their child’s hands or legs gently to remind the child not to move.
• The participants in the mock scanner were instructed as follows:
• "Now we’re going to show you a cartoon lasting 5 and a half minutes. Keep your head and body still while watching the cartoon and please keep your mind clear and do not fall asleep. Are you ready for this? Tell me when you’re ready."
• Children were praised if they performed well in controlling head movements during the mock scan, while those who performed less well were encouraged to try to keep their heads still in the next formal scan.

During the mock scan, prerecorded audio was played to simulate the noise of a real MRI scan. Head motion information collected in the mock scanner was presented in real time on the screen, and the main console recorded the head motion of the participant. A "red area" was shown on the screen when the participant’s head movement was greater than 0.2 mm. The head motion parameters were saved in the main console after the training session. More information about the MoTrak mock scanner can be found at the link https://www.nitrc.org/projects/motrak/. A total of 150 participants completed both mock and formal MRI scanning, consisting of 193 runs. After a quality check, 159 runs from 123 participants were kept for formal analysis, including 69 boys and 54 girls, of whom 87 participants completed one visit and 36 participants completed two visits.

### 2.3 Formal MRI data acquisition

Imaging data from CCNP-CKG were collected on a Siemens Trio 3 T scanner at Southwest University, Chongqing, China (Liu et al., 2021, Dong et al., 2020), and a total of 419 MRI scans from 196 participants were collected. For each visit, the scanning order was as follows: resting-state fMRI scan (rfMRI; 7 min 45 s), T1 magnetization prepared-rapid gradient echo (MP-RAGE; 8 min 19 s), and rfMRI scan (7 min 45 s), for a total of 15 min 30 s rfMRI scanning.

**Fig. 2:**
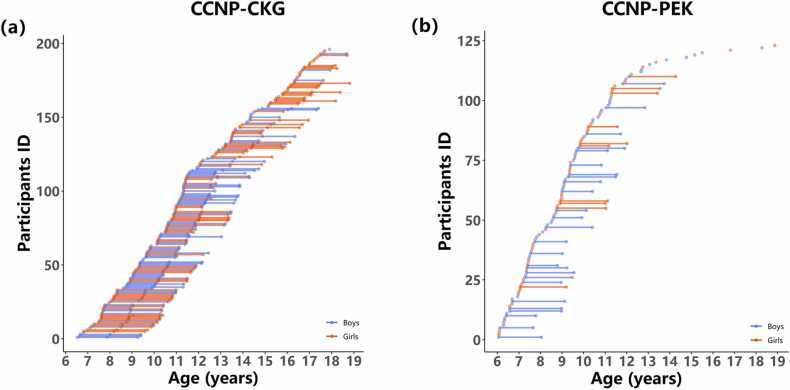
The sample demographics at CCNP-CKG (a) and CCNP-PEK (b).Participants’ ID is shown on the Y-axis, and their age at the time of scanning is indicated on the X-axis. Boys are indicated by the blue lines and dots, while the red lines and dots represent girls. The distance separating the two dots denotes the participant’s scan interval across trials.

Formal MRI scans at CCNP-PEK were performed after the mock training session and collected on a GE Discovery MR750 3 T scanner at the Institute of Psychology, Chinese Academy of Sciences, Beijing, China. A total of 159 MRI scans from 123 participants were collected. For each visit, the scanning order was as follows: rfMRI scan (6 mins), T1-weighted 3D spoiled gradient-recalled echo sequence (SPGR; 4 min 41 s), and rfMRI scan (6 mins), for a total of 12 mins rfMRI scanning. Detailed MRI scan parameters are listed in Table 1. The age and sex compositions of MRI scans are shown in Table 2 and Fig. 2.

**Table 1:** MRI scanning parameters at two imaging sites.

Site	Sequence	Resolution (mm^3^)	TR (ms)	TE (ms)	FOV (mm^2^)	FA (°)
CCNP-CKG	MPRAGE	1 × 1 × 1	2600	3.02	256	8
	EPI	3 × 3 × 3.33	2500	30	216	80
CCNP-PEK	SPGR	1 × 1 × 1	7	3	256	12
	EPI	3.5 × 3.5 × 4.2	2000	30	224	90

**Table 2:** The age and sex compositions of MRI scans at each CCNP site.

Site	Gender	Total number	Age-range	Mean of age	Sd of age
CCNP-CKG	91 M/105 F	196	6.52–17.91	11.02	2.69
CCNP-PEK	70 M/53 F	123	6.05–18.87	9.33	2.60

### 2.4 Data preprocessing

*Mock Data Preprocessing* Twenty-two scans from the original 193 scans were excluded from mock scanner training data for following reasons: participants did not complete the entire training, or the total length was complete but interrupted during training. Next, head movements were estimated in the order of (1) roll: rotation about the inferior to superior axis, (2) pitch: rotation about the right to left axis, (3) yaw: rotation about the anterior to posterior axis, (4) dS: displacement in the superior direction, (5) dL: displacement in the left direction and (6) dP: displacement in the posterior direction. The last three parameters are displacements and the first three parameters are rotation degrees in the three cardinal rotational directions. All head movements were calculated as the relative backward differences from the preceding time point.

*MRI Data Preprocessing* Anatomical images were first visually inspected, and nine participants were excluded for severe artifacts in T1 images due to excessive head motion. Then, the data were reorganized according to the BIDS (Gorgolewski et al., 2016) format and the MRI_QC toolkit was used to inspect the quality of rfMRI images. Next, MRI images were imported into the Connectome Computation System (CCS) for further analysis. The CCS pipeline is designed for multimodal MRI dataset preprocessing and is publicly available on GitHub (https://www.github.com/zuoxinian/CCS). The structural image went through the following preprocessing steps: (1) spatially adaptive nonlocal means denoising, (2) rough inhomogeneity correction, (3) spatial normalization into the MNI standard brain space, (4) inhomogeneity correction, (5) intensity normalization, (6) brain extraction by nonlocal intracranial cavity extraction (NICE), and (7) gray and white matter segmentation and surface reconstruction. The rfMRI image preprocessing included (1) dropping off the first 5 EPI volumes; (2) removing and interpolating temporal spikes; (3) correcting acquisition timing among image slices and head motion among image volumes, with the 3 displacements and 3 rotation head motions as described above also derived from the real rfMRI data; (4) normalizing the 4D global mean intensity to 10,000; (5) regressing out head motion artifacts and other spurious noise by using ICA-AROMA; and (6) removing linear and quadratic trends from the rfMRI signals to mitigate the scanner-related influences (Xing et al., 2022, Xu et al., 2015). In the current study, only motion data were extracted for further analysis and will be described in detail in the next section.

### 2.5 Framewise displacement estimation

Based on the abovementioned 3 rotation (roll, pitch and yaw) and 3 displacement (dS, dL and dP) parameters, two kinds of framewise displacement (FD) were calculated to assess head motion during rfMRI: root sum-square FD (RSS_FD) and absolute sum of FD (ABS_FD). RSS_FD was calculated as the root sum square of the 6 head movements, while ABS_FD was calculated as the absolute sum of the 6 head movements (Power et al., 2014). All the calculations were performed with the LFCD_IPN_computeMC function implemented in CCS. It has been reported that ABS_FD tends to be higher than RSS_FD. Here, to comprehensively evaluate head motion, both of these metrics were used for further analysis. Details of the head motion are listed in Table 3. After quality assurance, 159 mock scans from 123 participants were kept for further analysis.

**Table 3:** Details of head motion in two datasets.

FDtype	Site	Session	Mean	Min	Max	Standard deviation
ABS_FD^a^	CCNP-PEK	Mock^b^	0.31	0.02	4.37	0.57
ABS_FD	CCNP-PEK	Rest1^c^	0.18	0.06	0.60	0.19
ABS_FD	CCNP-PEK	Rest2^d^	0.25	0.06	0.17	0.28
ABS_FD	CCNP-CKG	Rest1	0.17	0.04	1.86	0.17
ABS_FD	CCNP-CKG	Rest2	0.09	0.02	1.01	0.18
RSS_FD^e^	CCNP-PEK	Mock	0.19	0.01	2.94	0.40
RSS_FD	CCNP-PEK	Rest1	0.10	0.03	0.37	0.11
RSS_FD	CCNP-CAS	Rest2	0.14	0.03	0.10	0.16
RSS_FD	CCNP-CKG	Rest1	0.16	0.03	1.94	0.16
RSS_FD	CCNP-CKG	Rest2	0.09	0.02	1.09	0.12

a Absolute sum of FD.

b The head motion value calculated from the mock scanner data.

c The head motion value calculated from the first fMRI scan data for each participant.

d The head motion value calculated from the second fMRI scan data for each participant which is the same visit with Rest1.

e Root-sum-square FD.

### 2.6 Head motion resampling

**Fig. 3:**
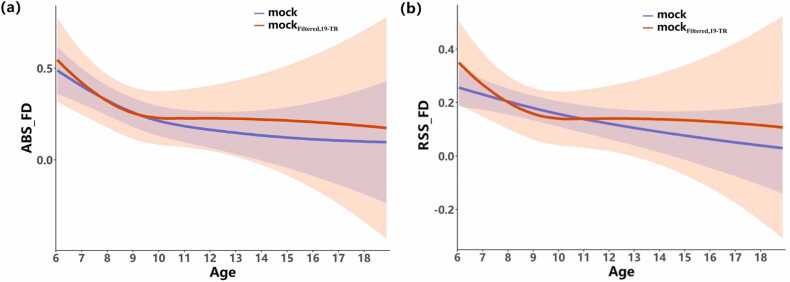
The growth and development trajectories of ABS_FD (a) and RSS_FD (b) based on the original TR and 19-TR in the mock scanner. Eq. (1) of the GAMM method was used to compute the age trajectories of the mock with TR (mock, blue line) and mock with 19-TR (mock_Filtered*,*19−TR_, red line).

Unlike in the formal scan session, the head motion in the mock scanner was collected by a sensor that was worn on the participant’s head and provided the position of the head relative to the transmitter. The original sampling rate (TR) of the system was 103 Hz, and the average buffering size was 11 samples, which resulted in a recording sampling rate of 9.285 Hz, much faster than that of the formal fMRI TR. It might be problematic to compare the head motion derived from fast-TR data directly to that from slow-TR data. FD is typically calculated by backward differences from the preceding time point, and in most studies, the TRs of fMRI are usually 2–4 s. In this study, the TR is 2 s in the formal rfMRI scan and 0.11 s in the mock scanner. To match the TR between mock and rfMRI sessions, ABS_FD and RSS_FD were also calculated by backward differences over 19 timepoints (i.e., 19 × 0.11 =2.09 s effective TR) and using position estimates that had been filtered to suppress dominant respiratory frequencies (0.2–0.5 Hz stop band) (Power et al., 2020), which are called ABS_FD_(Filtered,19−TR)_ and RSS_FD_(Filtered*,*19−TR)_. Both types of head motion in the mock scanner were calculated. The head motion difference between the original TR and 19-TR is shown in Fig. 3. In contrast to the previous studies (Power et al., 2014, Power et al., 2019, Power et al., 2020), the head movement calculated using the 19-TR interval was larger than those calculated using the original TR in the mock scanner in both ABS_FD and RSS_FD when the age range was 12–18. Here, the resampled head movements (19-TR interval) were utilized for further analysis to compare head motion in the mock scanner with two formal scan sessions.

### 2.7 Between-group comparison in head motion across age groups

Although it is generally acknowledged that head motion decreases with age during development, to the best of our knowledge, no prior study has systematically conducted research to reveal such a phenomenon. Thus, we first conducted a between-group analysis to explore whether such developmental differences could be captured at the group level. To achieve this, we divided the participants into two child groups and one teen group, and the cutoff age between the child and teen groups was consistent with our prior work (Dong et al., 2021), in which we observed that the transition from childhood to adolescence could be captured by the reorganization of the cortical network at approximately age 12. Since the sample size above 12 years old was quite small, we did not subdivide the teen group, but the children were divided into child1 (6−9) and child2 (9−12) groups. Repeated-measures ANOVA was conducted to examine the differences between age groups and scan sessions. We consider the differences between age groups to be the developmental characteristic of head motion, while the difference between scan sessions refers to the training effect, that is, whether head motion in formal scans was significantly reduced compared with the mock training session.

### 2.8 Construction of developmental trajectory

To quantify how head motion changes with age from childhood to adolescence, generalized additive mixed models (GAMMs) and generalized additive models for location, scale, and shape (GAMLSSs) were both employed. Subscript ‘mock’ is used to represent the head motion data collected in the mock scanner, ‘rest1’ is used to represent the head motion derived from the first rfMRI scan, and ‘rest2’ represents the head motion derived from the second rfMRI scan. Distinct from parametric general linear modeling, GAMM does not require a priori knowledge of the function form of the data, and a ‘smooth’ function term is applied to replace linear regressors. The nonlinear smooth function describes the optimal relationship between the covariates and the outcome variable of interest. For GAMM analyses on head motion, the main independent variable was age. A GAMM was implemented using the following formula in R language with the mgcv package (version 1.8–38):

(1) HM ∼ (age) + sex + (1 ∣ participant)

(2) HM ∼ (age) + sex + site + (1 ∣ participant)

HM refers to the head motion parameters (RSS_FD, ABS_FD, roll, pitch, yaw, dS, dL, dP in Eq. 1; mock, rest1, and rest2 in Eq. 2) and *s*() is a smoothing function, and the GAMM models head motion as a smoothing function of age. The GAMMs were first conducted to compare the developmental trajectories between the raw TR and resampling TR (i.e., 19-TR interval) for mock training data to determine which one was kept for further analysis. Then, GAMMs for each of the 6 head motion parameters and FD were conducted with the 19-TR interval.

According to the repeated-measures ANOVA and derived developmental trajectories, we found that the children below 9 years old moved significantly more than older children. To examine whether these younger children also benefited more from the mock training session, we then conducted GAMMs to depict the training effect during development. The training effect was defined as the head motion difference between the mock session and the formal session; for example, the head motion in the rest1 session was subtracted from that in the mock session, and we refer this difference as the benefits gained from the mock session, namely, the training effect. The training effects between mock and rest1 and mock and rest2 were then included in the GAMM to construct age-dependent trajectories.

To enlarge the sample size to build a robust and reliable developmental trajectory of head motion, we incorporated CCNP-CKG dataset in the study. Before combining the datasets, we first examined whether the head motion derived om the two sites exhibited similar developmental trends as in Eq. (2). The site was encoded as a nominal variable referring to CKG and PEK sites (CCNP-CKG=0, CCNP-PEK=1) in the GAMM, and sex was also considered as a covariate in Eq. (2). Finally, the GAMLSS, which has been employed by the World Health Organization (WHO) and Centers for Disease Control and Prevention (CDC) to build growth charts of height and weight for children (Borghi et al., 2006, Flegal and Cole, 2013, Organization et al., 2006), was applied to construct an overall developmental curve for head motion with a combination of both CCNP-CKG and CCNP-PEK datasets. Age, sex, and site were set as independent variables in the model, and the SHASH function was chosen to estimate head motion in the model. All GAMLSSs were conducted using the gamlss package (version 5.3–4) library in R (version 4.0.2) for Windows.

A series of GAMLSSs with different parameters were constructed: a Gaussian model with linear effects (Equation a nonlinear Gaussian homoscedastic model (Eq. 4), a nonlinear Gaussian heteroskedastic model (Eq. 5), and a non-Gaussian nonlinear heteroskedastic SHASH model(Eq. 6), and a nonlinear heteroskedastic SHASH model Jones and Pewsey (2009) (Eq. 7). The Akaike information criterion (AIC) was used for model selection.

The model with lowest AIC was chosen as the optimal model.

(3) HM ∼ (µ, σ), µ = *β*0 + *β*ageage + sex + site

(4) HM ∼ (µ, σ), µ = f(age) + sex + site

(5) HM ∼ (µ, σ), µ = f(age) + sex + site, *σ* = f*σ* (age) + sex + site

HM ∼ SHASH(µ, σ, *v*, τ), *μ* = f*μ*(age) + *sex* + *site*, *σ* = f*σ* (age) + sex + site,

(6) v = *βnu* + *sex* + *site*, τ = *β*tau + sex + site

HM ∼ SHASH(µ, σ, *v*, τ), µ = fµ(age) + *sex* + *site*, *σ* = f*σ* (age) + sex + site,

(7) v = fv (age) + *sex* + *site*, τ = fτ (age) + sex + site

Here, µ, *σ*, *v*, and *τ* refer to the location, scale, skewness, and kurtosis of the data distribution, respectively, which were the parameters of the function implemented in the GMALSS model. To better illustrate the differences in head motion between adult and pediatric populations, the ISYB dataset (Gao et al., 2022) consisting of 241 Chinese adult MRI images was used to demonstrate the head motion curve above 18 years old. We also constructed developmental curves separately for the untrained and trained groups. Notably, no training session was set before the rest1 session in CCNP-CKG dataset; thus, we combined the rest1 session in CCNP-PEK and mock session in CCNP-PEK to be the untrained group, while the rest1 session in CCNP-CKG and the rest1 session in CCNP-PEK were the trained group. Since we observed a rebound of head motion in younger children in the CCNP-PEK rest2 session, we inferred that this may reflect an exhaustion of the training effect; thus, the data from the rest2 session were not incorporated in the construction of the final developmental curves.

## 3 Results

### 3.1 Changes in head motion during school-age development

Each participant at the CCNP-PEK site was required to complete a training session in the mock scanner prior to the formal MRI scan at each visit, and each formal MRI scan consisted of two rfMRI scans; this kind of data collection provided the opportunity to investigate the impact of mock scanner training. In total, 87 children completed 1 visit scan, and 36 children completed 2 visits. Here, by leveraging children and adolescents who completed three sessions (i.e., mock, rest1, and rest2), we first demonstrated that younger children have significantly larger head movements than older children and, and benefited more from mock training. No significant difference was detected between age groups after training.

To directly demonstrate the age effect on head motion, we first conducted a between-group analysis. We divided the participants into three age groups: child1 (6–9 years old, 70 runs from 60 participants), child2 (9–12 years old, 68 runs from 44 participants), and teen (12–18 years old, 21 runs from 19 participants), and compared the head motions between scan sessions across these age groups. Repeated-measures ANOVA and post hoc analysis were performed on both ABS_FD and RSS_FD to examine whether younger children had significantly larger head movements. A significant difference was revealed between age groups (*F* = 5*.*48, degree of freedom (*df*) = 2, *p* = 2*.*81 × 10^−4^). The details of the post hoc analysis are listed in Table 4 and Table 5 for ABS_FD and RSS_FD, respectively. A significant difference in head motion was found between the child1 and child2 group (*p* = 0*.*03) in mock scanner measurements, revealing that children younger than 9 years old had larger head movements than children between 9 and 12 years old. No significant difference was found between age groups in the rest1 session, and the difference in head motion was reduced to 0.03 between the child1 and child2 groups, which was 0.1 in the mock session. A rebound of head motion in the child1 group was revealed in the rest2 session, and the difference from the teen group increased back to 0.09 (0.1 initially in the mock session), which was statistically significant (*p* = 0*.*03). We inferred that the rebound in head motion may reflect a limited effective period for mock training and a potential fatigue effect. The difference between the child2 and teen groups also increased to 0.04 while it was 0.01 in the initial mock session.

**Table 4:** Statistics of the post-hoc tests on the ABS_FD of age group with the different sessions.

Session	Group1	Group2	Difference (Group1-Group2)	Standard Error	p-value
Mock^a^	child1^b^	child2^c^	0.10	0.04	0.03 *
Mock	child 1	teen^d^	0.10	0.05	0.08
Mock	child 2	teen	0.01	0.06	0.98
Rest1^e^	child 1	child2	0.03	0.02	0.31
Rest1	child 1	teen	0.04	0.03	0.24
Rest1	child 2	teen	0.01	0.03	0.90
Rest2^f^	child 1	child2	0.05	0.03	0.14
Rest2	child 1	teen	0.09	0.04	0.03 *
Rest2	child 2	teen	0.04	0.04	0.60

a The head motion value calculated from the mock scanner data.

b Age range: 6–9 years old

c Age range: 9–12 years old

d Age range: 12–18 years old

e The head motion value calculated from the first fMRI scan data for each participant.

f The head motion value calculated from the second fMRI scan data for each participant which is the same visit with Rest1.

**Table 5:** Statistics of the post-hoc tests on the RSS_FD of age group with the different sessions.

Session	Group1	Group2	Difference (Group1-Group2)	Standard Error	p-value
Mock^a^	child1^b^	child 2^c^	0.05	0.08	0.04 *
Mock	child1	teen^d^	0.04	0.09	0.89
Mock	child2^c^	teen	-0.01	0.11	0.99
Rest1^e^	child1	child2	0.02	0.02	0.55
Rest1	child1	teen	0.05	0.03	0.12
Rest1	child2	teen	0.03	0.03	0.56
Rest2^f^	child1	child2	0.07	0.03	0.05
Rest2	child1	teen	0.09	0.04	0.78
Rest2	child2	teen	0.02	0.04	0.84

a The head motion value calculated from the mock scanner data.

b Age range: 6–9 years old

c Age range: 9–12 years old

d Age range: 12–18 years old

e The head motion value calculated from the first fMRI scan data for each participant.

f The head motion value calculated from the second fMRI scan data for each participant which is the same visit with Rest1.

Next, we examined the training effect by directly comparing the head motion between the training scan and formal scans. The results in Table 6 show that evident improvement was achieved in the rest1 session revealed by ABS_FD.

**Table 6:** Statistics of the post-hoc tests on the session with the different FD type.

FD	Session1	Session2	Difference (Session1- Session2)	Standard Error	p-value
ABS_FD^a^	Mock^b^	Rest1^c^	0.10	0.02	1.89 × 10–6 ***
ABS_FD	Mock	Rest2^d^	0.11	0.06	0.13
ABS_FD	Rest1	Rest2	0.01	0.04	0.94
RSS_FD^e^	Mock	Rest1	0.12	0.02	8.17 × 10–6 ***
RSS_FD	Mock	Rest2	0.09	0.01	9.56 × 10–10 ***
RSS_FD	Rest1	Rest2	-0.03	0.02	0.14

a Absolute sum of FD

b The head motion value calculated from the mock scanner data.

c The head motion value calculated from the first fMRI scan data for each participant.

d The head motion value calculated from the second fMRI scan data for each participant which is the same visit with Rest1.

e Root sum-square FD

(*p* = 1*.*89 × 10^−6^), while RSS_FD revealed a significant improvement in both the rest1 (*p* = 8*.*17 × 10^−6^) and rest2 sessions (*p* = 9*.*56 × 10^−10^). No difference was found between the rest1 and rest2 sessions. Together, these between- group comparisons clearly demonstrate an evident age effect in head motion, that is, children younger below 9 years old exhibited larger movements than those older participants, and a brief mock training significantly improved their performance in the following formal scan, but a rebound of head motion in the rest2 session indicated that the duration of mock training needs to be carefully considered in future studies.

**Fig. 4:**
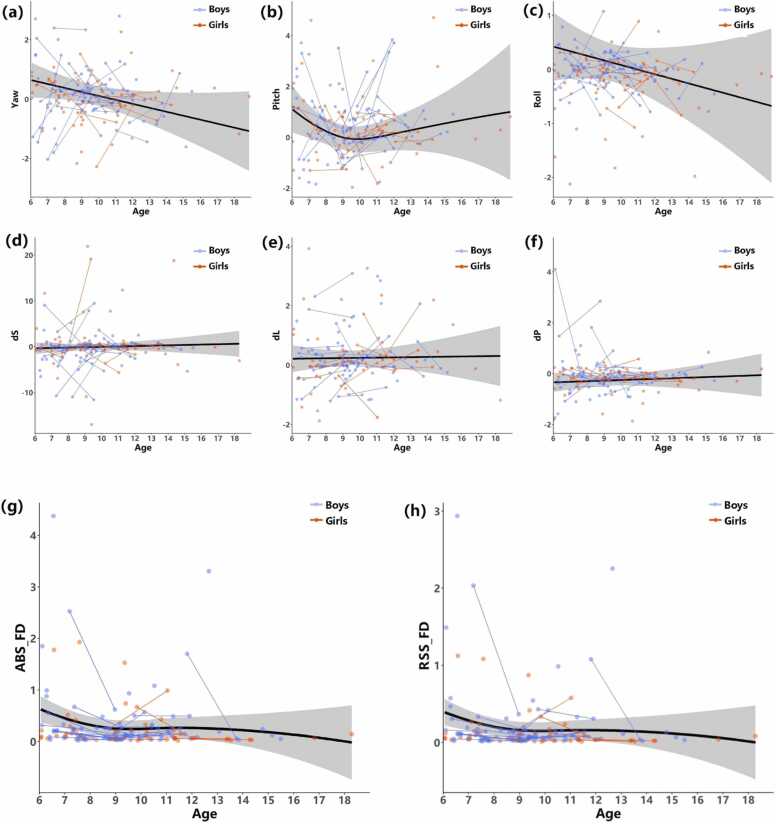
The growth curves with the age of six head motion parameters (a-f) and two types of head motion (g and h) in mock scanner data modeled by Eq. (1) using the GAMM method. The blue dots and lines indicate boys, and the red dots and lines indicate girls. The black lines show the trajectories in the child and adolescent groups and are surrounded by shaded 95% confidence intervals (gray region). In particular, before the age of 9, ABS_FD showed a downward trend, and the decline rate decreased with increasing age after 9 years old. Yaw: rotation about the anterior to posterior axis, Pitch: rotation about the right to left axis, Roll: rotation about the inferior to superior axis, dS: displacement in the superior direction, dL: displacement in the left direction, dP: displacement in the posterior direction.

Although it is clear from the between-group analysis that mock training effectively reduced head motion in formal scans, it is still unclear that whether it reached the commonly applied criteria and to what extent a brief mock scanner training would help to save more participant data in neuroimaging studies. To further depict how the head motion changes after mock training, a GAMM was constructed to generate continuous developmental cures for six head motion direction parameters (yaw, pitch, roll, dS, dL, and dP) and two types of head motion (RSS_FD and ABS_FD) calculated by CCS, which were modeled individually. The details of the GAMM are listed in Eq. (1), and the results were shown in Fig. 4. Both RSS_FD and ABS_FD declined from 6 years old to 9 years old, and after age 9, the head motion became quite stable, indicating that younger children (*<* 9 years old) need the training more than older children. Since few participants above 12 years old were included, the changing pattern during adolescence needs to be further investigated in future studies. The developmental curves of 3 rotational and 3 translational parameters are displayed in Fig. 4. Independent samples t tests revealed no gender difference in head motion (*t* = 0*.*16*, p* = 0*.*056*, df* = 151). The developmental pattern of pitch movements tended to be associated with the FD, and yaw and roll movements quickly decreased with age, while the translational parameters all slightly increased with age.

**Fig. 5:**
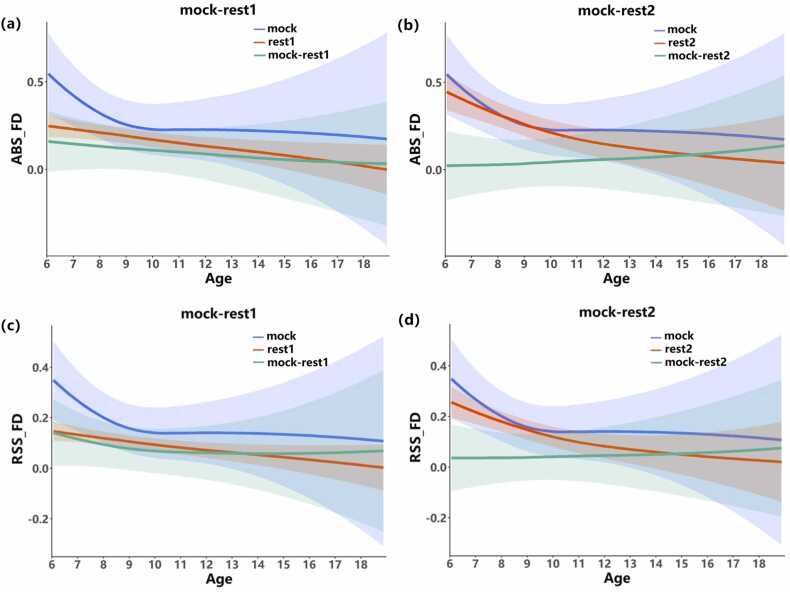
The growth and development trajectories of mock vs. rest1 (left) and mock vs. rest2 (right) based on ABS_FD (Panels a and b) and RSS_FD (Panels c and d) in the CCNP_PEK site. Note that data from rest1 and rest2 were in one fMRI session. Eq. (1) of the GAMM method was used to compute the age trajectories of the mock (blue line) and rest (red line). The difference between mock and rest (green line) depicting the head motion trajectory (mock-rest) was used for the assessment of age-related changes in head motion.

As revealed by the above between-group analysis, younger children benefit more from mock training, and we further built continuous developmental curves of head movements to demonstrate this phenomenon. GAMMs were constructed separately for mock, rest1, and rest2 sessions, and the practice effect was modeled as the subtraction between mock and formal scanning. As shown in Fig. 5, the decreasing tendency of head motion in mock training indicates that although the head movements rebounded in the rest2 session, they were still less than those in the mock session, especially in participants older than 8–9 years old (green line in Panels b and d). The rebounds of head motion in the rest2 session were greater in children younger than 10 years old, during which the head motion also declined faster than in older children. This may be accounted for by the fatigue effect; the younger children are, the harder it is to maintain focus and self-control during scanning.

Through the head motion in the mock scan training session, it was observed that head motion showed a decreasing trend with age. This trend was also observed in the rest1 session. To compare the head movement in mock and rest1, the difference between mock and rest1 was modeled by the GAMM method. The difference also showed a trend of decreasing with age, and the curve of the difference between mock and rest1 was larger than 0, which also indicated that the head motion during the rest1 session was less than that in the mock session, revealing a potential training effect.

**Fig. 6:**
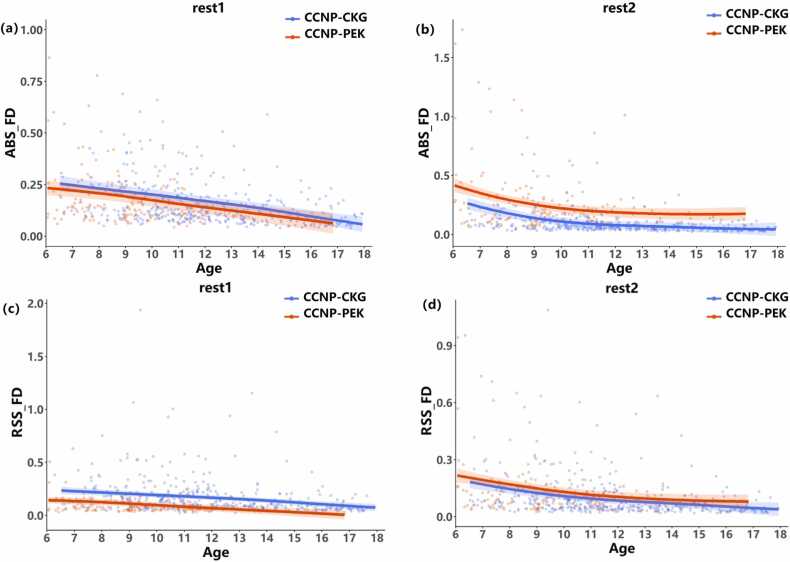
Comparison of head motion of the first fMRI scan between the two CCNP sites. The blue line and dots indicate that the data come from CCNP-CKG, while the red line and dots indicate that the data come from CCNP-PEK. The trajectories are built by Eq. (2) of the GAMM method and surrounded by shaded 95% confidence intervals. (a) and (c) are based on ABS_FD, while (b) and (d) are based on RMS_FD.

Then, the head motion data from the CCNP-CKG site were included to validate the effect of the mock scanner training. The first fMRI scanning data (rest1) from the two different sites were compared between two groups, as the subjects at CCNP-CKG (untrained group, 196 participants,age range = 6*.*52 ∼ 17*.*91, mean age =11.02) had not undergone a pretraining session before the formal scan; thus, we hypothesized that the participants at the CCNP-PEK site (trained group, 123 participants,age range = 6*.*05 ∼ 18*.*87, mean age = 9.33) would have smaller head movements compared with those at CCNP-CKG in the first scan session. The growth development trajectories of the two groups were constructed using Eq. (2) with the GAMM. As shown in Fig. 6(a) and (b), regardless of ABS_FD or RSS_FD, the head motion trajectory of the untrained group was greater than that of the trained group, and there was a significant difference between the two groups in the RSS_FD by independent sample t test (*p* = 7*.*36 ×10^−6^*, t* = −3*.*83). Growth curves of ABS_FD and RSS_FD for the head motion in the second fMRI scanning data (rest2) are shown in Fig. 6(c) and (d). The head motion in CCNP-CKG participants was less than that in CCNP-PEK participants and there was a significant difference between the two groups in the ABS_FD test by independent sample t test (*p* = 1*.*20 × 10^−6^*, t* = −3*.*83). When comparing ABS_FD at the two sites of participants aged 9 − 12 years, the difference was not significant (*p* = 0*.*08). One possible reason is that the rest2 session was the ‘third’ scan for CCNP-PEK participants, causing them to become more tired, resulting in more head motion. We emphasize that training with a mock scanner before the formal scan could significantly reduce head movements in both children and adolescents.

### 3.2 Comparison of head motion between children and adults

It is clear from previous findings that head motion decreases with age, and at approximately 9–10 years old, it reaches a stable point. To further compare with the adult population, we included the Chinese adult dataset (I See Your Brain, ISYB, (http://www.doi.org/10.11922/sciencedb.00740) (Gao et al., 2022)), which contains multimodal neuroimaging data from 215 right-handed healthy Chinese volunteers, to investigate the motion differences between children and adults. After the CCS pipeline estimated head motion for the ISYB dataset, a total of 26 participants were excluded according to the widely applied threshold of FD (0*.*2 mm); however, to comprehensively depict head motion across age stages, we kept these subjects in the current study.

**Fig. 7:**
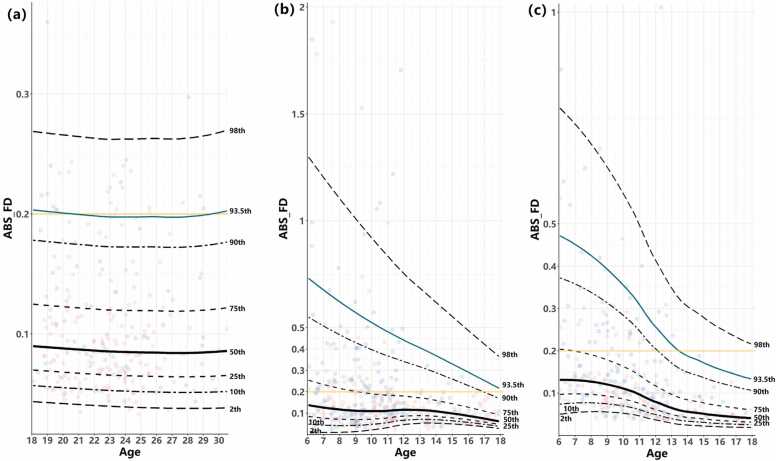
The head motion growth charts of adults (a) and younger people (b refers to the untrained group and c refers to the trained group) modeled by the GAMLSS method. Head motion data from the ISYB database must pass the data quality check, and ABS_FD was calculated by the CCS pipeline. Blue dots represent males, and red dots represent females; different black lines represent different centiles, as marked on the right. The 93.5^th^ centile, which is suggested as a threshold of head motion in the younger population, is represented by the green lines.

GAMLSS was used to model the developmental trajectory of head motion data from 241 young adults aged 18–30 years. The AIC results are shown in Table 7 (ISYB dataset). The model with the lowest AIC was chosen as the optimal model (Model 1). The 2, 10^*th*^, 25^*th*^, 50^*th*^, 75^*th*^, 90^*th*^, and 98^*th*^ percentiles of adult head motion were plotted the same time (Fig. 7a, black line). The 0.2 mm FD threshold is indicated as the yellow line in Fig. 7(a). We found that this criterion corresponded closely to the 93*.*5^*th*^ centile of the head motion chart for young adults in the ISYB dataset (Fig. 7a, green line). Based on the head motion growth chart built by GAMLSS and the comparison of AIC (Table 7, CCNP dataset), the optimal threshold was Model 1. We then labeled the same 93*.*5^*th*^ centile curves of the untrained group (mock in CCNP-PEK and rest1 in CCNP-CKG) and the trained group (rest1 in CCNP-PEK and rest2 in CCNP-CKG) in the CCNP dataset (Fig. 7b and c, green lines). As demonstrated in the figure, the 93*.*5^*th*^ line in the adult group remained stable across ages; however, in the children and adolescent groups, the 93*.*5^*th*^ line varied.

**Table 7:** The AIC of different GAMLSS model in head motion of two different dataset.

Dataset	Model1	Model2	Model3	Model4	Model5
ISYB	-791.65	-789.24	-789.24	-786.07	-783.81
CCNP	-599.74	-598.99	-594.42	-569.63	-569.59

dramatically during development, and the head motion reached approximately 0.6–0.7 mm at 6 years old, which is almost 3 times that in the adult group. It was not until 12 years old that the head motion decreased to 0.5 mm. If the single criterion was applied to the initial untrained participants, more participants would be excluded from the study.

## 4 Discussion

Head motion during scanning remains a critical challenge in fMRI studies, particularly in young children, aging adults, and clinical populations. Head motion causes well-recognized artifacts in neuroimaging, which can be especially problematic for the interpretation of resting-state studies (Power et al., 2012). In addition, the presence of motion- related artifacts dynamically changes with age. To reduce excessive head movement, training with a mock scanner prior to formal scanning has been proposed. However, the mock scanner’s efficacy has yet to be formally quantified. In this study, we primarily focused on the pediatric population. By leveraging head motion data from one mock scan and two formal scans from the Chinese Color Nest Cohort dataset (Liu et al., 2021), we generated growth curves of head motion under several conditions (with or without mock training). Our goal was to systemically evaluate the training effect of the mock scanner in pediatric populations and estimate the extent to which a reduction in head movements can be suppressed by an additional training session.

### 4.1 Effective mock scanner training

At the CCNP-PEK site, a prescan with a mock scanner was set up before formal MRI data collection, providing opportunity to estimate the head movements in the mock training session. For the head motion data in the mock scanner and two formal rest sessions, repeated-measures ANOVA and developmental trajectories were conducted to explore the effectiveness of mock scanner training as well as to identify which age stage benefited the most from mock scanner training. Consistent with a previous study (Frew et al., 2022), the largest head movements occurred in the pitch direction and dominated the integrated head motion trajectory. The effect of mock scanner training was evident from between-group comparisons, greatly improving the performance in formal scans, and young children aged from 6 to 9 years benefited the most from mock training (Table 2). Although the cartoon movie (Peppa Pig) and the decorative style of the mock scanner were more aimed at children, which may partly account for the greater improvement in younger children, it was still evident that adolescents benefited from the pretraining session (see Fig. 5); their head movements decreased in the formal scan (green line above zero indicating decrease in head motion). Approximately 75% of participants above 12 years old were capable of controlling their head movements below 0.2 mm (Fig. 7b), while this number increased to 90% after mock training (Fig. 7c), which is much closer to that in the adult group (Figs. 7a, 93.5% centile). Another possibility is that a ceiling effect may exist in adolescents since an inflation point of head motion trajectory was revealed at 12 years old (Fig. 5, Fig. 7b), indicating that adolescents are intrinsically more capable of keeping their heads still. In summary, according to our results, we recommend that warm and relaxed styles are preferred as priorities for mock scanner and room decoration, and any exciting factors should be avoided during mock scanner training.

### 4.2 Developmental changes of head motion

Having established that mock training effectively reduces head movements in the young children group, we then calculated the developmental trajectory of head motion to demonstrate how it changes with age and the corresponding training effect. Our result is consistent with that of a previous study (Slifer et al., 1994) showing that head motion generally decreased with age at a more rapid rate before approximately 9 years old (Fig. 4 g and h). In addition to physical immaturity, psychological factors may also account for this phenomenon; children generally do not talk about their own internal mental experiences as readily as adults or with the same level of observation or introspection (Wellman and Hickling, 1994). Children have also been shown to have different relationships between mental time travel and the use of cognitive resources compared to adults (Ye et al., 2014), and these developmental differences in mind wandering could have significant effects on head motion. Children will not engage in mind wandering in an immersive or sustained manner in the absence of a task; as such, they may become restless much more quickly (Frew et al., 2022).

The greatest improvement in head motion by mock training also occurred in the participants younger than 9 years old (Fig. 5, green line). Similar results have been reported previously: the rate of good quality structural MRI scans of children in the age range of 4–7 years was 81/90 using a similar mock scanner training protocol (Hallowell et al., 2008), and for children younger than 7 years, we obtained a success rate of 30/43 in fMRI studies (De Bie et al., 2010). In this study, we performed a continuous evaluation of head movements with age rather than assigning a discrete score to the scan; moreover, our results provide potential guidance on the age range of the population that needs the most training. A rebound of head movements was revealed in the second formal scan of the participants under 9 years old at CCNP-PEK site. We inferred that this may be partly due to fatigue since the participants had undergone 5 and a half minutes of training in addition to formal scans (6 min). It has been reported that a longer-term scan is accompanied by larger head motion (Engelhardt et al., 2017). This indicates that the duration of the training scan should be taken into consideration to avoid fatigue and potential irritation in younger participants. Nonetheless, the improvement of head motion by a mock MRI scanner training protocol is obvious and applicable in the pediatric population.

### 4.3 Pediatric charts on head motion

Head movement is a physiological trait, and children and adolescents have more body movements than adults, yet overall head movement decreases with age in developmental samples (Engelhardt et al., 2017). If the same criterion absolute movements (e.g., FD) for head motion is applied to both adults and children, more younger children or clinical populations are likely to be excluded due to excessive head movements (Nebel et al., 2022). To characterize the proportion of participants with certain head movements across age ranges, we conducted growth chart analysis for adult and pediatric populations. Growth charts boast three invaluable benefits: enhancing public health, screening the developmental status of individuals, and monitoring their abnormal growth as an early detection tool (Scherdel et al., 2016). The establishment of the growth curve for head motion not only provides a comprehensive understanding of dynamic changes with age but also indicates the percentiles of specific head movements at different ages. For fMRI studies, especially among pediatric and clinical populations, a single threshold is widely used to determine the exclusion criterion, resulting in possible selection bias and data loss or resource waste (Zeng et al., 2014). In this study, we evaluated the number of participants excluded if a single head motion threshold derived from adults was applied to the pediatric population.

We used the adult ISYB brain imaging database (Gao et al., 2022), which is collected at the same site as CCNP-PEK, as the reference. The growth charts of head movements in children, adolescents (Figs. 7b and 7c), and adults (Fig. 7a) were established. It is worth noting that the head movement trajectory of the 18–30-year-old adult population remained relatively stable as age increases but showed a downward trend with age in children and adolescents. Furthermore, for adults, due to the stability of the head motion development trajectory, it is reasonable to use a specific threshold as the quality assurance criterion, only around 6.5% of adult participants are excluded with a criterion of 0.2 mm FD. However, for untrained children and adolescents, this number increases to 25% at age 6 (black bold line in Fig. 7) and does not decrease until 18 years old, where it reaches 10%. It was revealed that 0.2 mm FD stably corresponds to the 93*.*5^*th*^ percentile line in the adult group but dynamically changes from the 75^*th*^ to 90^*th*^ percentile line from 6 years old to 17 years old in the untrained population. However, this number increased to the 98^*th*^ percentile line after mock training (Fig. 7c), revealing that more participants were conserved. Heavy head motion induces artificial results, such as long-range functional connectivity (Poldrack et al., 2002, Dijk et al., 2012); thus, strict quality assurance in head motion is essential for reliable and valid findings. However, excessive exclusion of participants, especially in pediatric population, may cause a huge loss in sample size and selection bias. Although no perfect solution exists for the current situation, the mock training protocol provides an acceptable strategy, and significant improvements in controlling head motion as well as conservation of sample size are achieved with one additional mock session. We believe that it is particularly promising in pediatric neuroimaging studies, especially for large-scale population-level projects, to utilize the mock training session to improve data quality and experimental efficiency.

## 5 Limitations

Our results revealed that a brief mock scanner training maybe beneficial in helping young children control their head movements during subsequent scans. However, it is important to validate this finding in future studies using larger samples. The sample size above 15 years old is limited in the current study, and the stable head movements observed here may be largely affected by the small sample size. Accumulated data in the future would help to provide more comprehensive details in the teenage group. It is also important for future studies to evaluate the effect of training duration, and limit the possible fatigue during subsequent formal scanning, reaching a balance between time cost and effectiveness in desirable head motion control. In addition, fixation during the real scan probably helps the participants keep their head still, and future studies should further evaluate to what extent the visual materials would affect the training effect. For setting up a more efficient mock scanner protocol, it is crucial to further evaluate how head movements are improved with training duration and whether an optimal parameter is in common use for all age ranges.

## 6 Conclusions

Head movement has a significant impact on the quality of fMRI data, and the data disturbed by heavy head movements should be excluded from the analysis to ensure the reliability and validity of scientific findings. Here, by evaluating head movements with and without prior mock training, we found that 5 min of mock scanner training is effective at reducing head motions among children and adolescents, and younger children under 9 years old benefit the most. We then further estimated the percentage of participants conserved with the single head motion criterion derived from the adult population. The results revealed that with mock training, approximately 10% of participants were conserved at 13 years old, and this number increased to 18.5% at 6 years old. Given the significant improvement in motion control, we proposed that an additional mock scanner training should be included in fMRI research prior to formal scanning for better data quality and larger samples.

## CRediT authorship contribution statement

**Peng Gao:** Writing - Original draft preparation and revision, Methodology, Software. **Yin-Shan Wang:** Data Preparation, Writing - Revision. **Qiu-Yu Lu:** Writing - Revision. **Meng-Jie Rong:** Writing - Revision. **Xue-Ru Fan:** Data Preparation, Writing - Revision. **Avram J. Holmes:** Writing - Revision. **Hao-Ming Dong:** Concept of design, Writing - Original draft preparation and revision, Methodology, Software. **Hai-Fang Li:** Concept of design, Writing - Revision. **Xi-Nian Zuo:** Concept of design, Writing, Methodology, Software.

## Declaration of Competing Interest

The authors declare that they have no known competing financial interests or personal relationships that could have appeared to influence the work reported in this paper.

## References

[bib1] Alexander L.M., Escalera J., Ai L., Andreotti C., Febre K., Mangone A., Vega Potler N., Langer N., Alexander A., Kovacs M. (2017). An open resource for transdiagnostic research in pediatric mental health and learning disorders. Sci. Data.

[bib2] Alexander Bloch A., Clasen L., Stockman M., Ronan L., Lalonde F., Giedd J., Raznahan A. (2016). Subtle in-scanner motion biases automated measurement of brain anatomy from in vivo MRI. Hum. Brain Mapp..

[bib3] Bjork J.M., Straub L.K., Provost R.G., Neale M.C. (2017). The ABCD study of neurodevelopment: Identifying neurocircuit targets for prevention and treatment of adolescent substance abuse. Curr. Treat. Options Psychiatry.

[bib4] Bookheimer S.Y. (2000). Methodological issues in pediatric neuroimaging. Ment. Retard. Dev. Disabil. Res. Rev..

[bib5] Borghi E., de Onis M., Garza C., Van den Broeck J., Frongillo E.A., Grummer Strawn L., Van Buuren S., Pan H., Molinari L., Martorell R. (2006). Construction of the World Health Organization child growth standards: selection of methods for attained growth curves. Stat. Med..

[bib6] Bullmore E.T., Brammer M.J., Rabe Hesketh S., Curtis V.A., Mcguire P.K. (2015). Methods for diagnosis and treatment of stimulus-correlated motion in generic brain activation studies using fmri. Hum. Brain Mapp..

[bib7] Caballero Gaudes C., Reynolds R.C. (2017). Methods for cleaning the bold fMRI signal. Neuroimage.

[bib8] Carolina M., Lepage M., Evans A.C. (2019). Head motion: the dirty little secret of neuroimaging in psychiatry. J. Psychiatry Neurosci..

[bib9] Casey B.J., Cannonier T., Conley M.I., Cohen A.O., Barch D.M., Heitzeg M.M., Soules M.E., Teslovich T., Dellarco D.V., Garavan H. (2018). The adolescent brain cognitive development (abcd) study: imaging acquisition across 21 sites. Dev. Cogn. Neurosci..

[bib10] Cgyab C., Bc B., Ck C., Sc A., Rccb D., Adm C., Ql B., Xnz E., Fxca C., Mpma B. (2013). A comprehensive assessment of regional variation in the impact of head micromovements on functional connectomics. NeuroImage.

[bib11] Ciric R., Wolf D.H., Power J.D., Roalf D.R., Baum G.L., Ruparel K., Shinohara R.T., Elliott M.A., Eickhoff S.B., Davatzikos C. (2017). Benchmarking of participant-level confound regression strategies for the control of motion artifact in studies of functional connectivity. Neuroimage.

[bib12] Davidson M.C., Thomas K., Casey B. (2003). Imaging the developing brain with fMRI. Ment. Retard. Dev. Disabil. Res. Rev..

[bib13] De Bie H., Boersma M., Wattjes M.P., Adriaanse S., Vermeulen R.J., Oostrom K.J., Huisman J., Veltman D.J. (2010). Preparing children with a mock scanner training protocol results in high quality structural and functional MRI scans. Eur. J. Pediatr..

[bib14] Di Martino A., Yan C.G., Li Q., Denio E., Castellanos F.X., Alaerts K., Anderson J.S., Assaf M., Bookheimer S.Y., Dapretto M. (2014). The autism brain imaging data exchange: towards a large-scale evaluation of the intrinsic brain architecture in autism. Mol. Psychiatry.

[bib15] Dijk K., Sabuncu M.R., Buckner R.L. (2012). The influence of head motion on intrinsic functional connectivity MRI. Neuroimage.

[bib16] Dong H.M., Margulies D.S., Zuo X.N., Holmes A.J. (2021). Shifting gradients of macroscale cortical organization mark the transition from childhood to adolescence. Proc. Natl. Acad. Sci..

[bib17] Dong H.M., Castellanos F.X., Yang N., Zhang Z., Zhou Q., He Y., Zhang L., Xu T., Holmes A.J., Yeo B.T. (2020). Charting brain growth in tandem with brain templates at school age. Sci. Bull..

[bib18] Dosenbach N., Koller J.M., Earl E.A., Miranda Dominguez O., Klein R.L., Van A.N., Snyder A.Z., Nagel B.J., Nigg J.T., Nguyen A. (2017). Real-time motion analytics during brain MRI improve data quality and reduce costs. NeuroImage.

[bib19] Engelhardt L.E., Roe M.A., Juranek J., DeMaster D., Harden K.P., Tucker Drob E.M., Church J.A. (2017). Children’s head motion during fmri tasks is heritable and stable over time. Dev. Cogn. Neurosci..

[bib20] Flegal K.M., Cole T.J. (2013). Construction of LMS parameters for the Centers for Disease Control and Prevention 2000 growth charts. Natl. Health Stat. Report.

[bib21] Frew S., Samara A., Shearer H., Eilbott J., Vanderwal T. (2022). Getting the nod: pediatric head motion in a transdiagnostic sample during movie-and resting-state fMRI. PLoS One.

[bib22] Friston K.J., Williams S., Howard R., Frackowiak R.S., Turner R. (1996). Movement-related effects in fMRI time-series. Magn. Reson. Med..

[bib23] Gao P., Dong H.M., Liu S.M., Fan X.R., Jiang C., Wang Y.S., Margulies D., Li H.F., Zuo X.N. (2022). A chinese multi-modal neuroimaging data release for increasing diversity of human brain mapping. Sci. Data.

[bib24] Gorgolewski K.J., Auer T., Calhoun V.D., Craddock R.C., Das S., Duff E.P., Flandin G., Ghosh S.S., Glatard T., Halchenko Y.O. (2016). The brain imaging data structure, a format for organizing and describing outputs of neuroimaging experiments. Sci. Data.

[bib25] Greene D.J., Koller J.M., Hampton J.M., Wesevich V., Van A.N., Nguyen A.L., Hoyt C.R., McIntyre L., Earl E.A., Klein R.L. (2018). Behavioral interventions for reducing head motion during MRI scans in children. Neuroimage.

[bib26] Hallowell L.M., Stewart S.E., de Amorim e Silva C.T., Ditchfield M.R. (2008). Reviewing the process of preparing children for MRI. Pediatr. Radiol..

[bib27] Harms M.P., Somerville L.H., Ances B.M., Andersson J., Barch D.M., Bastiani M., Bookheimer S.Y., Brown T.B., Buckner R.L., Burgess G.C. (2018). Extending the Human Connectome Project across ages: Imaging protocols for the lifespan development and aging projects. Neuroimage.

[bib28] Jolly E., Sadhukha S., Chang L.J. (2020). Custom-molded headcases have limited efficacy in reducing head motion during naturalistic fMRI experiments. NeuroImage.

[bib29] Jones M.C., Pewsey A. (2009). Sinh-Arcsinh distributions. Biometrika.

[bib30] Kotsoni E., Byrd D., Casey B. (2006). Special considerations for functional magnetic resonance imaging of pediatric populations. J. Magn. Reson. Imaging.

[bib31] Krause F., Benjamins C., Eck J., Lührs M., van Hoof R., Goebel R. (2019). Active head motion reduction in magnetic resonance imaging using tactile feedback. Hum. Brain Mapp..

[bib32] Liu S., Wang Y.S., Zhang Q., Zhou Q., Cao L.Z., Jiang C., Zhang Z., Yang N., Dong Q., Zuo X.N. (2021). Chinese Color Nest Project: An accelerated longitudinal brain-mind cohort. Dev. Cogn. Neurosci..

[bib33] Mascarell Maričić L., Walter H., Rosenthal A., Ripke S., Quinlan E.B., Banaschewski T., Barker G.J., Bokde A.L., Bromberg U., Büchel C. (2020). The IMAGEN study: a decade of imaging genetics in adolescents. Mol. Psychiatry.

[bib34] McKay D.R., Knowles E.E., Winkler A.A., Sprooten E., Kochunov P., Olvera R.L., Curran J.E., Kent J.W., Carless M.A., Göring H.H. (2014). Influence of age, sex and genetic factors on the human brain. Brain Imaging Behav..

[bib35] Murphy K., Fox M.D. (2017). Towards a consensus regarding global signal regression for resting state functional connectivity MRI. Neuroimage.

[bib36] Nebel M.B., Lidstone D.E., Wang L., Benkeser D., Mostofsky S.H., Risk B.B. (2022). Accounting for motion in resting-state fMRI: what part of the spectrum are we characterizing in autism spectrum disorder?. Neuroimage.

[bib37] Nooner K.B., Colcombe S.J., Tobe R.H., Mennes M., Benedict M.M., Moreno A.L., Panek L.J., Brown S., Zavitz S.T., Li Q. (2012). The NKI-Rockland sample: a model for accelerating the pace of discovery science in psychiatry. Front. Neurosci..

[bib38] Organization W.H. (2006). WHO child growth standards based on length/height, weight and age. Acta Paediatr. Suppl..

[bib39] Pardoe H.R., Hiess R.K., Kuzniecky R. (2016). Motion and morphometry in clinical and nonclinical populations. Neuroimage.

[bib40] Poldrack R.A., Paré Blagoev E.J., Grant P.E. (2002). Pediatric functional magnetic resonance imaging: progress and challenges. Top. Magn. Reson. Imaging.

[bib41] Power J.D., Barnes K.A., Snyder A.Z., Schlaggar B.L., Petersen S.E. (2012). Spurious but systematic correlations in functional connectivity mri networks arise from subject motion. Neuroimage.

[bib42] Power J.D., Mitra A., Laumann T.O., Snyder A.Z., Schlaggar B.L., Petersen S.E. (2014). Methods to detect, characterize, and remove motion artifact in resting state fMRI. Neuroimage.

[bib43] Power J.D., Lynch C.J., Silver B.M., Dubin M.J., Martin A., Jones R.M. (2019). Distinctions among real and apparent respiratory motions in human fMRI data. Neuroimage.

[bib44] Power J.D., Lynch C.J., Dubin M.J., Silver B.M., Martin A., Jones R.M. (2020). Characteristics of respiratory measures in young adults scanned at rest, including systematic changes and “missed” deep breaths. Neuroimage.

[bib45] Pruim R.H., Mennes M., van Rooij D., Llera A., Buitelaar J.K., Beckmann C.F. (2015). ICA-AROMA: a robust ica-based strategy for removing motion artifacts from fMRI data. Neuroimage.

[bib46] Raschle N.M., Lee M., Buechler R., Christodoulou J.A., Chang M., Vakil M., Stering P.L., Gaab N. (2009). Making MR imaging child’s play-pediatric neuroimaging protocol, guidelines and procedure. J. Vis. Exp..

[bib47] Rosenberg D.R., Sweeney J.A., Gillen J.S., Kim J., Varanelli M.J., O’HEARN K.M., Erb P.A., Davis D., Thulborn K.R. (1997). Magnetic resonance imaging of children without sedation: preparation with simulation. J. Am. Acad. Child Adolesc. Psychiatry.

[bib48] Salat D.H., Buckner R.L., Snyder A.Z., Greve D.N., Desikan R.S., Busa E., Morris J.C., Dale A.M., Fischl B. (2004). Thinning of the cerebral cortex in aging. Cereb. Cortex.

[bib49] Salimi Khorshidi G., Douaud G., Beckmann C.F., Glasser M.F., Griffanti L., Smith S.M. (2014). Automatic denoising of functional MRI data: combining independent component analysis and hierarchical fusion of classifiers. Neuroimage.

[bib50] Satterthwaite T.D., Wolf D.H., Loughead J., Ruparel K., Elliott M.A., Hakonarson H., Gur R.C., Gur R.E. (2012). Impact of in-scanner head motion on multiple measures of functional connectivity: relevance for studies of neurodevelopment in youth. Neuroimage.

[bib51] Savalia N.K., Agres P.F., Chan M.Y. (2017). Motion-related artifacts in structural brain images revealed with independent estimates of in-scanner head motion. Hum. Brain Mapp.

[bib52] Savalia N.K., Agres P.F., Chan M.Y., Feczko E.J., Kennedy K.M., Wig G.S. (2017). Motion-related artifacts in structural brain images revealed with independent estimates of in-scanner head motion. Hum. Brain Mapp..

[bib53] Scherdel P., Dunkel L., van Dommelen P., Goulet O., Salaün J.F., Brauner R., Heude B., Chalumeau M. (2016). Growth monitoring as an early detection tool: a systematic review. Lancet Diabetes Endocrinol..

[bib54] Slifer K.J., Bucholtz J.D., Cataldo M.D. (1994). Behavioral training of motion control in young children undergoing radiation treatment without sedation. J. Pediatr. Oncol. Nurs..

[bib55] Slifer K.J., Cataldo M.F., Cataldo M.D., Llorente A.M., Gerson A.C. (1993). Behavior analysis of motion control for pediatric neuroimaging. J. Appl. Behav. Anal..

[bib56] Smyser C.D., Snyder A.Z., Neil J.J. (2011). Functional connectivity MRI in infants: exploration of the functional organization of the developing brain. Neuroimage.

[bib57] Thompson W.K., Hallmayer J., O’Hara R., Initiative A.D.N. (2011). Design considerations for characterizing psychiatric trajectories across the lifespan: application to effects of apoe-*ε* 4 on cerebral cortical thickness in Alzheimer’s disease. Am. J. Psychiatry.

[bib58] Van Dijk K.R., Hedden T., Venkataraman A., Evans K.C., Lazar S.W., Buckner R.L. (2010). Intrinsic functional connectivity as a tool for human connectomics: theory, properties, and optimization. J. Neurophysiol..

[bib59] Van Essen D.C., Smith S.M., Barch D.M., Behrens T.E., Yacoub E., Ugurbil K., Consortium W.M.H. (2013). The WU-Minn Human Connectome Project: an overview. Neuroimage.

[bib60] Wellman H.M., Hickling A.K. (1994). The mind’s “I”: children’s conception of the mind as an active agent. Child Dev..

[bib61] Xing X.X., Xu T., Jiang C., Wang Y.S., Zuo X.N. (2022). Connectome Computation System: 2015-2021 updates. Sci. Bull..

[bib62] Xu T., Yang Z., Jiang L., Xing X.X., Zuo X.N. (2015). A Connectome Computation System for discovery science of brain. Sci. Bull..

[bib63] Ye Q., Song X., Zhang Y., Wang Q. (2014). Children’s mental time travel during mind wandering. Front. Psychol..

[bib64] Zeng L.L., Wang D., Fox M.D., Sabuncu M., Hu D., Ge M., Buckner R.L., Liu H. (2014). Neurobiological basis of head motion in brain imaging. Proc. Natl. Acad. Sci..

[bib65] Zuo X.N., Xu T., Milham M.P. (2019). Harnessing reliability for neuroscience research. Nat. Hum. Behav..

[bib66] Zuo X.N., He Y., Betzel R.F., Colcombe S., Sporns O., Milham M.P. (2017). Human Connectomics across the Life Span. Trends Cogn. Sci..

